# Marine Benthic Cyanobacteria Contain Apoptosis-Inducing Activity Synergizing with Daunorubicin to Kill Leukemia Cells, but not Cardiomyocytes

**DOI:** 10.3390/md8102659

**Published:** 2010-10-14

**Authors:** Linn Oftedal, Frode Selheim, Matti Wahlsten, Kaarina Sivonen, Stein Ove Døskeland, Lars Herfindal

**Affiliations:** 1 Department of Biomedicine, University of Bergen, Jonas Lies vei 91, 5009 Bergen, Norway; E-Mails: linn.oftedal@biomed.uib.no (L.O.); frode.selheim@biomed.uib.no (F.S.); stein.doskeland@biomed.uib.no (S.O.D.); 2 Proteomic Unit at the University of Bergen, Jonas Lies vei 91, 5009 Bergen, Norway; 3 Department of Food and Environmental Sciences, University of Helsinki, P. O. Box 56, 00014 Helsinki, Finland; E-Mails: matti.wahlsten@helsinki.fi (M.W.); kaarina.sivonen@helsinki.fi (K.S.); 4 Translational Signalling group, Haukeland Univ. Hospital, Jonas Lies vei 91, 5009 Bergen, Norway

**Keywords:** AML, cyanobacteria, apoptosis, cancer, leukemia

## Abstract

The potential of marine benthic cyanobacteria as a source of anticancer drug candidates was assessed in a screen for induction of cell death (apoptosis) in acute myeloid leukemia (AML) cells. Of the 41 marine cyanobacterial strains screened, more than half contained cell death-inducing activity. Several strains contained activity against AML cells, but not against non-malignant cells like hepatocytes and cardiomyoblasts. The apoptotic cell death induced by the various strains could be distinguished by the role of caspase activation and sensitivity to the recently detected chemotherapy-resistance-associated prosurvival protein LEDGF/p75. One strain (M44) was particularly promising since its activity counteracted the protective effect of LEDGF/p75 overexpressed in AML cells, acted synergistically with the anthracycline anticancer drug daunorubicin in AML cells, and protected cardiomyoblasts against the toxic effect of anthracyclines. We conclude that culturable benthic marine cyanobacteria from temperate environments provide a promising and hitherto underexploited source for novel antileukemic drugs.

## 1. Introduction

Marine cyanobacteria have been recognized as a source for new drugs and drug leads [1,2]. For instance, clinical anticancer trials are being conducted with soblidotin (TZT-1027) and synthadotin (ILX-651), two derivatives of dolastatin 10 isolated from the benthic tropical marine cyanobacteria *Symploca* sp. [3,4]. In addition, derivatives of curacin A isolated from the benthic tropical marine cyanobacteria *Lyngbya majuscula* are in preclinical trials for treatment of cancer [5]. Most cytotoxic marine cyanobacterial compounds have been isolated from tropical benthic *Lyngbya* sp. and *Symploca* sp. forming large mats, whereas only a few cytotoxic compounds have been reported from other marine genera [1,2,6]. Less studied marine cyanobacteria genera should therefore be included in anticancer screens.

Acute myeloid leukemia (AML) is the second most common form of leukemia [7]. Treatment of AML consists mainly of chemotherapy using cytosine arabinoside (Ara-C) and the anthracycline daunorubicin [8]. Chemoresistant disease, either as primary resistance to induction therapy or as relapse due to resistant residual disease, is the major cause of treatment failure in most cancers, including AML [9,10]. There are several recently discovered factors involved in chemoresistance. Increased expression of the transcriptional co-activator Lens Epithelium-Derived Growth Factor LEDGF/p75 has been reported in chemotherapy-resistant human AML blasts as well as in breast and bladder carcinomas [11–13]. Another anti-apoptotic protein that confers chemotherapy resistance to AML cells, and is associated with a poor therapeutic prognosis, is Bcl-2 [14,15]. There is a need for new therapies in treatment of AML, since if standard therapy fails there is usually no alternative treatment [9,10]. New therapies are also needed for other types of cancer, and drugs used to treat leukemia have often been transferred to other cancers with little or no modifications, as exemplified by the anthracyclines [16].

We have previously shown that cyanobacteria constitute a rich source of bioactivities [17–21], and wanted to investigate if culturable marine benthic cyanobacteria from temperate environments were potential producers of compounds with anticancer potential. We investigated strains sampled from the shores of the Baltic Sea and found that a high proportion of these induced cell death in AML cells. Three strains were able to induce apoptosis in chemoresistant AML cells with enforced expression of LEDGF/p75, whereas non-malignant cells remained viable. In combination with daunorubicin these three anti-AML activities induced an apoptotic synergistic response in leukemia cells, but not in cardiomyoblasts.

## 2. Results

### 2.1. Screening of benthic marine cyanobacteria for leukemia cell death inducers

**Figure S1:**
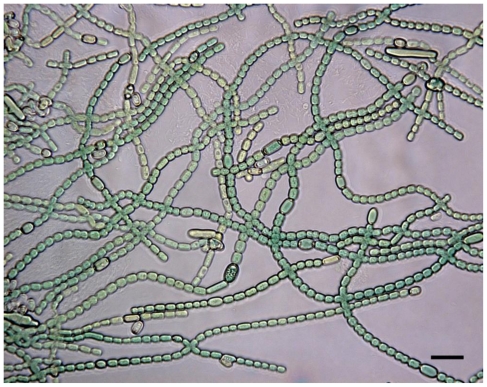
The microscopic appearance of the *Anabaena* strain M30 in culture. This appearance is representative of all the benthic *Anabaena* strains studied. The bar represents 20 μm.

**Figure 1:**
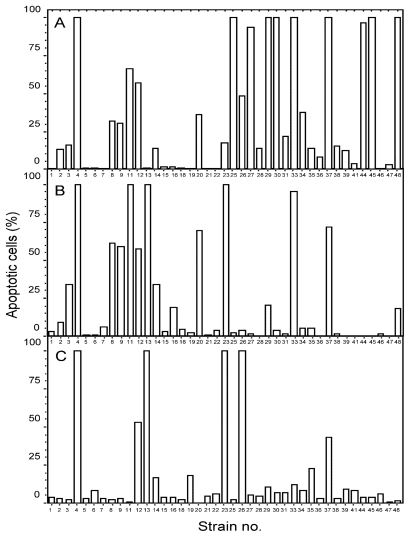
Induction of leukemia cell death by cyanobacterial extracts. Rat acute myeloid leukemia cells were incubated with aqueous (**A**), aqueous methanol (**B**) or methanol-dichloromethane (**C**) cyanobacterial extracts at a concentration of 4 mg DW biomass/mL for 18 hours before being fixed in 2% buffered formaldehyde (pH 7.4) with the DNA-dye Hoechst 33342. Cell death was assessed by microscopic assessment of surface and nuclear morphology. The results are the mean, n = 3. Vehicle treated cells had less than 2% apoptosis.

Forty-one benthic Baltic Sea cyanobacterial strains of the genera *Anabaena*, *Nostoc*, *Calothrix* and *Cyanothece* (Table S1 and Figure S1), previously screened for death-inducing activity against primary hepatocytes [17], were extracted sequentially with water, aqueous 70% methanol, and 1:1 methanol:dichloromethane (A, B and C extracts, respectively). They were first screened for cell death-inducing activity against the AML cell line IPC-81wt [22], which originates from the BNML rat model, known to be a reliable predictor of the outcome of anticancer treatment [23]. Nineteen A extracts, 13 B extracts, and 6 C extracts contained cell death inducers. Ten strains (M4, 8, 9, 11, 12, 13, 23, 26, 33, 37) contained cell death inducing activity in more than one extract, like strain M26 that contained activity in the A and C extract, but not in the intermediate B extract (Figure 1). Several of the extracts with anti-AML activity, most notably the A extracts from the *Anabaena* sp. M4, M27, M30, M44 and M45 (Figure 1), were previously found to lack activity against normal primary hepatocytes (see [17]). We therefore conclude that marine benthic cyanobacteria are not only a rich source of cell death inducing activity, but also contain apoptogens selective for AML cells.

### 2.2. Modulation of blood platelet activity by cyanobacterial extracts

**Figure 2:**
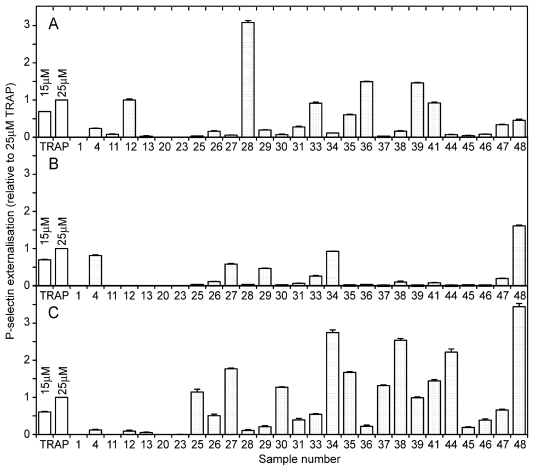
Modulation of blood platelet activity by cyanobacterial extracts. Gel-filtered human blood platelets were incubated for 30 min with PE-conjugated anti-CD62P and aqueous (**A**), aqueous methanol (**B**) or methanol-dichloromethane (**C**) cyanobacterial extracts, 4 mg DW biomass/mL, or thrombin receptor agonist peptide (TRAP). The platelets were then fixed and analyzed for externalization of CD62P by flow cytometry. The results are mean and SEM, n = 3–9.

Thrombocyte function is critical in leukemia, which is characterized by platelet deficiency due both to thrombocytopenia and thromboembolic complications [8,24,25]. Moreover, treatment of leukemia with cytotoxic drugs may change platelet function *in vivo* (reviewed in [26]). We therefore studied if any of the cyanobacterial extracts contained blood platelet activating activity that could limit usefulness as anti-AML agents. Interestingly, we found that a high proportion of the C extracts contained blood platelet activators (Figure 2). Of more importance for the present study is that the antileukemic A extracts of M27, M30, M44 and M45, unlike the anticancer drug daunorubicin [26], lacked platelet-modulating activity (Figure 2A).

### 2.3. Drug-relevant features of the apoptogens in cyanobacteria M27, M30 and M44

**Figure 3:**
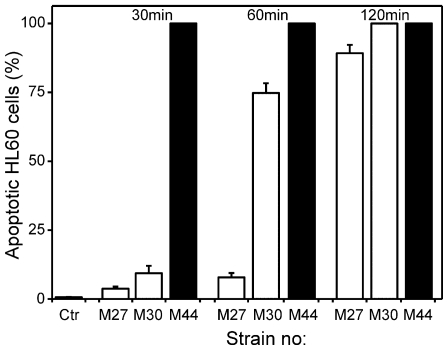
Cyanobacterial extracts induced rapid apoptosis in HL-60 acute myeloid leukemia cells. HL-60 cells were incubated without (Ctr) or with 4 mg DW biomass/mL of aqueous cyanobacterial extracts for 30, 60 or 120 min. Cells were fixed and stained, and apoptosis assessed as described in the legend to Figure 1. The results are mean and SEM, n = 3.

Although the IPC-81 AML cells used in the primary screen respond in a clinically relevant way to present day anti-leukemic drugs both *in vivo* and *in vitro* [23,27], it is important to know if the IPC-81 apoptogens induce death in other relevant AML cell lines. In this respect, the aqueous extracts from M27, M30 and M44 were overall more efficient than those from M45 and M4 (not shown). M27, M30 and M44 induced apoptosis rapidly in human HL-60 AML cells (Figure 3), even compared to a higher-than-therapeutic concentration of daunorubicin in these cells ([27], data not shown). The high potency and rapid action of M27, M30 and M44 towards the leukemia cells are advantageous for *in vivo* therapy, since there will be a shorter burst of the drug, thus allowing less time for the formation of potentially generally harmful metabolites of the drug. For these reasons, the aqueous extracts from M27, M30 and M44 were selected for further studies.

**Figure 4:**
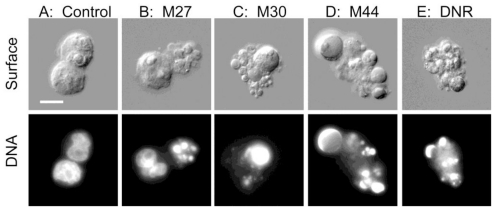
Apoptotic morphology induced by cyanobacterial extracts in AML cells. Rat leukemia cells (**A**: control) were added the aqueous extract of the cyanobacterial strain M27 (**B**), M30 (**C**), M44 (**D**) or 50 nM daunorubicin (DNR, **E**) as described in the legend to Figure 1, and photomicrographs were taken using differential interference contrast (upper) and UV (lower). Bar represent 10 μm.

Cell death induced by M27, M30 or M44 had the morphological hallmarks of apoptosis, such as chromatin condensation, nuclear fragmentation, surface budding and shedding of apoptotic bodies (Figure 4A–D). The apoptotic phenotype was as striking as the one observed in cells incubated with the benchmark anti-AML drug daunorubicin (Figure 4E).

**Figure 5:**
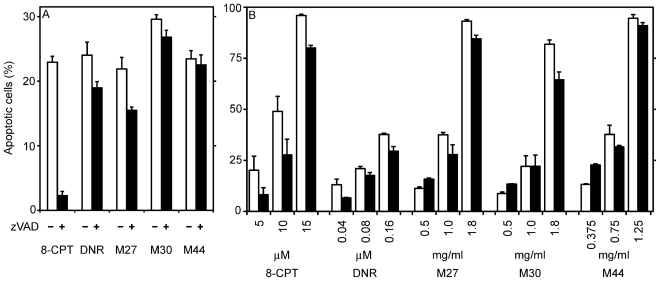
Involvement of caspases and LEDGF/p75 in apoptosis induced by cyanobacterial extracts. (**A**) Rat acute myeloid leukemia cells were incubated with or without 50μM of the pan-caspase inhibitor zVAD for 30 min before addition of 200 nM daunorubicin (DNR), 25 μM 8-chlorophenylthio-cAMP (8-CPT), or 4 mg DW biomass/mL of cyanobacterial extracts. The experiments were stopped after 7 h of incubation. zVAD-fmk alone induced less than 3% apoptosis during the 7.5 h of incubation. (**B**) Rat acute myeloid leukemia cells with or without enforced expression of the survival factor LEDGF/p75 (black and white columns, respectively) were incubated with various concentrations of known apoptosis-inducers or cyanobacterial extracts for 18 hours. The cells were fixed and stained, and apoptosis assessed as described in the legend to Figure 1. The results are mean and SEM, n = 3–6.

Cells overexpressing Bcl-2 are resistant towards stimuli leading to mitochondrial instability with release of pro-apoptotic proteins [28]. IPC-81 AML cells with enforced expression of Bcl-2 [29] were resistant towards apoptosis induced by either daunorubicin or by the apoptogens in M27, M30 or M44 (not shown). This indicates that like daunorubicin, the cyanobacterial apoptogens signaled through the mitochondrial cell death pathway. Significant differences were discovered, however, between the AML apoptogens with respect to the involvement of caspase dependent cell death pathways. The apoptosis induced by activation of the cAMP-dependent protein kinase by a cAMP analogue was completely abrogated by the caspase inhibitor zVADfmk, whereas apoptosis induced by M30 or M44 appeared to be caspase independent and apoptosis induced by daunorubicin or M27 was partially inhibited by the caspase inhibitor (Figure 5A).

Drug resistance is the major cause of therapeutic failure in AML [9,10]. We have found that LEDGF/p75 is consistently upregulated in AML blasts from patients with chemoresistant AML [11]. LEDGF/p75 is a survival factor reported to inhibit a caspase-independent lysosomal cell death pathway [13]. As previously observed [11], we found, that IPC-81 cells with enforced expression of LEDGF/p75 were less sensitive towards apoptosis induced by either cAMP analog or daunorubicin. In contrast, such cells had intact sensitivity towards M44, and even showed enhanced apoptosis, compared to wild-type cells, at low M44 concentrations (Figure 5B). M27 and M30 induced a response intermediate between M44 and daunorubicin. They resembled M44 by tending to enhance apoptosis in the LEDGF/p75 overexpressing cells at low concentrations, but were like daunorubicin less efficient against these cells at high concentrations (Figure 5B). We conclude that M44 and to a lesser extent M27 and M30 had better ability than daunorubicin to circumvent the chemoresistance provided by LEDGF/p75.

**Figure 6:**
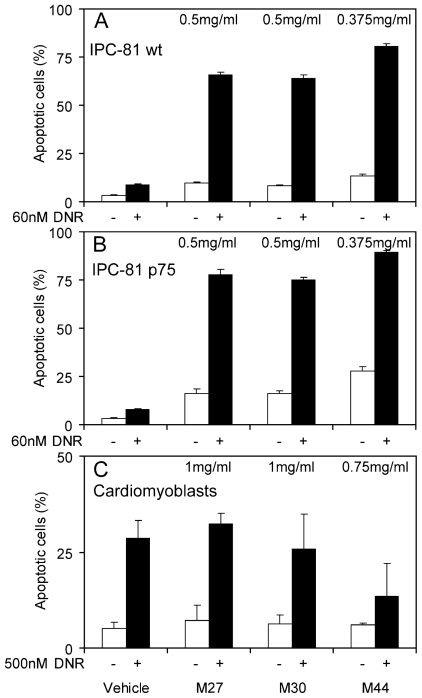
Cyanobacterial extracts induce a synergistic apoptotic response with daunorubicin in acute myeloid leukemia cells, but not in cardiomyoblasts. Rat acute myeloid leukemia cells without (**A**) or with enforced expression of the survival factor LEDGF/p75 (**B**), or cardiomyoblasts (**C**), were incubated for 18 hours with cyanobacterial extracts alone or in combination with daunorubicin (DNR) (white and black columns, respectively) before fixation, staining and assessment of apoptosis as described in the legend to Figure 1. The results are mean and SEM, n = 3.

Since M44, and to a lesser extent M27 and M30, differed from daunorubicin in cell death mechanism and in efficiency against LEDGF/p75 overexpressing cells, they could be useful adjuncts to daunorubicin. In fact, all three synergized strongly with daunorubicin to induce apoptosis whether the AML cells had enforced expression of LEGDF/p75 or not (Figure 6A and B). This suggests that the cyanobacterial apoptogens have potential to enhance the efficiency of daunorubicin, also in cells expressing the chemoresistance-associated LEDGF/p75.

A major limitation for drugs and drug combinations is unwanted side effects [10,16]. Since the major side effect of anthracyclines in cancer therapy is cardiotoxicity [8,16], we studied whether any of the cyanobacterial activities had intrinsic cardiomyoblast toxicity or enhanced the toxicity of daunorubicin in such cells. The intrinsic ability of M27, M30 or M44 to induce cardiomyoblast death was far lower than for the AML cells, confirming their AML selectivity (Figure 6 and data not shown). Furthermore, at concentrations effective against AML cells, M27, M30 and M44 failed to enhance the daunorubicin toxicity towards cardiomyoblasts. On the contrary, M44 significantly reduced the daunorubicin toxicity (Figure 6C).

## 3. Discussion

We have previously reported that cyanobacteria from temperate environments are a rich source of bioactivities [17–21]. In the present study we have shown that culturable marine benthic cyanobacteria from temperate environments produce bioactive compounds with potential as anticancer drugs. In line with previous studies [17,20,21], we found that cyanobacteria of the genera *Anabaena* most frequently contained bioactivities.

Based on the initial screen we focused on the five aqueous cyanobacterial extracts, which induced apoptosis preferentially in AML cells (Figure 1), whereas normal primary hepatocytes remained viable [17]. Three of them (M30, M27 and M44) induced apoptosis in human AML cells in less than two hours, suggesting an efficient and direct cell death pathway. The rapid action is an advantage from a pharmaceutical standpoint, especially if the active compounds should have short biological half-life, whether due to renal excretion or metabolic conversion to inactive metabolites.

As based on defining morphological criteria [30], the cyanobacteria induced classical apoptotic cell death (Figure 4). The death depended little or not at all on caspases (Figure 5A). From a drug point of view it is interesting that the apoptogenic activity in M27, M30 and M44 was high also against AML cells with enforced expression of LEDGF/p75 (Figure 6B), which is upregulated in most cases of chemotherapy-resistant AML [11].

It is recommended that new AML treatment strategies should consist of drug combinations [10] to cover a broader range of cell death pathways, since acquired apoptotic resistance caused by deregulation of death signaling mechanisms is a common feature in malignant cells [31]. If two compounds act synergistically their combination may allow lower doses of each and thereby decrease the toxicity as compared to monotherapy. We found that the combination of a moderate concentration of the anticancer drug daunorubicin with M27, M30 or M44 (Figure 6A) induced a synergistic apoptotic response in AML cells (Figure 6A) and chemoresistant AML cells with enforced expression of LEDGF/p75 (Figure 6B). This means that these cyanobacterial apoptogens have the ability to greatly improve the therapeutic index of daunorubicin. A severe side effect of anthracycline therapy is cardiotoxicity [8,16] and it is crucial that anticancer drug combinations do not escalate this cardiotoxic effect. Notably, neither of the cyanobacterial extracts synergized with daunorubicin causing cardiomyoblast toxicity (Figure 6C). In fact, M44 appeared to protect the cardiac cells against daunorubicin-induced toxicity. These properties of the cyanobacterial apoptogens are important in AML drug development, since less toxic treatment strategies as well as strategies to circumvent chemoresistance are needed [10].

**Figure S2:**
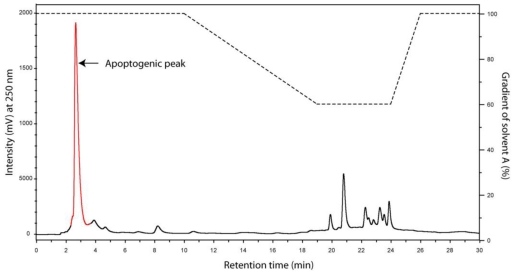
RP-HPLC separation of the analytes in the apoptotogenic aqueous extract of cyanobacterial strain M44. The sample (60 μL) prepurified by solid phase extraction (see above) was further purified by chromtaography on a column (Aquasil C18; 3 × 150 mm, Thermo Hypersil-Keystone) coupled to a Merck-Hitachi LaChrom HPLC system (VWR, West Chester, USA). The mobile phases were 10mM TEAF pH 3 (A), and 100% methanol (B). The flow rate was 0.5 mL/min and monitoring wavelength was 250nm. The bioactivity eluted in fraction 2.–3.8 min (red).

We found that the majority of the anti-AML-activity resided in the aqueous extracts. This is promising since high aqueous solubility is considered pharmacokinetically favorable [32,33]. On the other hand, highly water-soluble compounds can be difficult to isolate using high performance liquid chromatography, as illustrated for the death-inducing activity of M44 (supplementary Figure S2). Recent advances in scaled-up counter-current chromatography [34,35] and further improvements of large scale culturing of benthic cyanobacteria will presumably pave the way for more satisfactory large scale isolation of the highly polar anticancer compounds from marine benthic and other [20] cyanobacteria.

## 4. Conclusions

In summary, this study shows that culturable marine benthic cyanobacteria are a promising source of bioactive compounds with potential as anticancer drug candidates or lead compounds. The observation that compounds from benthic cyanobacteria combined with daunorubicin induce a synergistic apoptotic response in AML cells, but not in normal cardiomyoblasts supports this conclusion. The apoptogens contained in these marine benthic cyanobacteria, coupled to current progress in scaled-up culturing and chemical purification, indicate that this hitherto underexploited niche of cyanobacteria is an exploitable source for novel anticancer drug candidates.

## 5. Experimental Section

### 5.1. Materials

Dichloromethane, dimethylsulfoxide (DMSO), Hoechst 33342, penicillin/streptomycin, 8-chlorophenylthio-cAMP and daunorubicin were from Sigma-Aldrich (St. Louis, USA). Methanol and formaldehyde were from VWR (West Chester, USA). Dulbecco’s Modified Eagle Medium (DMEM), Fetal Calf Serum (FCS) and Horse Serum (HS) were supplied by EuroClone® Life Sciences Division (Milan, Italy). RPMI 1640 Medium (Gibco®invitrogen cell culture) was from Invitrogen AS (Carlsbad, USA). PE-conjugated anti-human CD62P antibody (CAT. #348107) was from Becton Dickinson Biosciences (San Jose, USA). Thrombin receptor agonist peptide (TRAP, SFLLRN) was from the Biotechnology centre of Oslo (Oslo, Norway). Z-val-ala-DL-asp-fluoromethylketone (zVAD-fmk) was from Alexis Biochemicals (San Diego, USA).

### 5.2. Isolation and cultivation of cyanobacterial strains

The cyanobacterial strains used in this study [17] were collected from sediment, sand, surface of stones, rocks, water plants, macroalgae, mussels or molluscs from the Baltic Sea at Porkkala (Gulf of Finland). Strains had previously been identified [17] based on morphology [36] as *Anabaena*, *Nostoc*, *Calothrix* or *Cyanothece* (see supplementary material: Figure S1 and Table S1) and mass cultivated as previously described [17]. Cyanobacterial cells were cultured for 20–60 days before they were harvested by centrifugation and freeze dried.

### 5.3. Sequential extraction of cyanobacterial biomass

Extraction of cyanobacterial biomass was performed as described by Herfindal *et al.* [17]. In brief, freeze-dried cyanobacterial biomass was first extracted in water, followed by extraction in 70% aqueous methanol and finally 1:1 (v/v) methanol:dichloromethane. The extracts were then dried in a centrifugal vacuum concentrator (Savant SpeedVac®AES1010) and resuspended in either 0.2 mL water (aqueous; A extract), 0.05 mL water (70% aqueous methanol; B extract) or 0.05 mL DMSO (methanol:dichloromethane; C extract).

### 5.4. Maintenance of cell lines, experimental conditions and determination of cell death

The rat promyeloid leukemia cell lines IPC-81 wild-type cells [22], and IPC-81 with enforced expression of LEDGF/p75 [11] or Bcl-2 [29], were cultured in Dulbecco’s modified Eagle’s medium (DMEM) supplemented with 50 U/mL of penicillin and 0.05 mg/mL streptomycin and 10% (v/v) heat inactivated horse serum. The rat cardiomyoblastic cell line H9c2 (ATCC: CRL-1446) was cultured in DMEM with antibiotics as described above but enriched with 10% (v/v) heat inactivated fetal calf serum. When the cells reached 80% confluence, they were detached by mild trypsin treatment (0.33 mg/mL trypsin for 5 min at 37 °C), washed and reseeded in fresh medium to 25% confluence. The human myeloid leukemia cell line HL-60 (ATCC: CCL-240) was cultured in RPMI medium with antibiotics as described above and enriched with 10% (v/v) heat inactivated fetal calf serum. All cells were cultured at 37 °C in a humid atmosphere of 5% CO_2_.

For assays of cell death, the suspension cells were centrifuged at 160 × g for 4 min, resuspended in fresh medium and 1.5 × 10^4^ cells were seeded in 0.1 mL in a 96-well tissue culture plate. The cardiomyoblasts were detached, centrifuged, and 2000 cells were seeded in each well of a 96-well tissue culture plates 24 hours before start of the cell death assays. The experiments were stopped by addition of 0.1 mL PBS containing 4% formaldehyde (pH 7.4) and 0.01 mg/mL of the DNA specific fluorescent dye Hoechst 33342. Apoptosis was scored by differential interference contrast and fluorescence microscopy (Axiovert 35M, Zeiss) as previously described [37]. Untreated cells or cells added vehicle exhibited a viability of more than 97%.

### 5.5. Isolation, incubation and flow cytometry analysis of human blood platelets

Isolation of human blood platelets was from fresh whole blood, as previously described [38,39]. In brief, platelet rich plasma obtained by centrifugation of whole blood was gel filtered and platelets eluted into a calcium-free Tyrode’s buffer containing glucose and bovine serum albumin at pH 7.3.

The gel-filtered human blood platelets (3.5 × 10^7^ per sample) were incubated under non-stirring conditions for 30 min with cyanobacterial extract or Thrombin Receptor Agonist Peptide SFLLRN (TRAP), PBS (5 μL) and anti-CD62PE (0.24 μg/mL) in a total volume of 25 μL. The platelets were then fixed 1:16 with 0.5% (v/v) paraformaldehyde in PBS and analysis of P-selectin (CD62) expression was carried out using a FACSort flow cytometer as described by Selheim *et al.* [40]. Ten thousand platelets, identified by size (light scatter gating), were analyzed for CD62PE fluorescence per sample.

## Supplementary material

Table S1. Code numbers and genera of the cyanobacteria included in this study.

**Table:** 

Code number	Genus
1	*Nostoc sp.*
2	*Anabaena sp.*
3	*Anabaena sp*
4	*Anabaena sp*
5	*Calothrix sp*
6	*Nostoc sp.*
7	*Nostoc sp.*
8	*Anabaena sp.*
9	*Anabaena sp.*
11	*Anabaena sp.*
12	*Anabaena sp.*
13	*Anabaena sp.*
14	*Anabaena sp.*
15	*Calothrix sp*
16	*Calothrix sp*
18	*Calothrix sp*
19	*Calothrix sp*
20	*Anabaena sp.*
21	*Calothrix sp*
22	*Nostoc sp.*
23	*Anabaena sp.*
25	*Anabaena sp*
26	*Anabaena sp*
27	*Anabaena sp*
28	*Nostoc sp*
29	*Anabaena sp*
30	*Anabaena sp.*
31	*Anabaena sp*
33	*Anabaena sp*
34	*Cyanothece sp.*
35	Unidentified
36	*Nostoc sp.*
37	*Anabaena sp.*
38	*Cyanothece sp.*
39	*Anabaena sp*
41	*Nostoc sp.*
44	*Anabaena sp*
45	*Anabaena sp*
46	*Anabaena sp*
47	*Calothrix sp*
48	*Anabaena sp*

### Bioguided Partial Purification of the Apoptogenic Constituent in the Aqueous M44-Extract

#### Solid phase extraction

The residue of the dried water extract from 3 mg original dry weight was dissolved in 0.15 mL water, added 0.6 mL methanol, and next 0.15 mL chloroform. The mixture was vortexed, added 0.45 mL water, vortexed again, and centrifuged at 6000 × g for 2 min at room temperature. The upper aqueous/methanol phase contained the bioactivity, and was collected, evaporated, and dissolved in 1 mL of 10% aqueous methanol. This sample was applied to a mixed mode reversed phase and strong anion exchanger (Oasis®MAX 186000367, Waters Corporation, Milford, USA) solid phase extraction (SPE) cartridge, conditioned with 3mL methanol and equilibrated with 3 mL of 10% aqueous methanol. After sample application the column was washed with another 3 mL of 10% aqueous methanol. The bioactivity appeared in the combined flow through and wash fractions, which was evaporated and redissolved in 60 μL of aqueous 10mM Triethylamine formic acid (TEAF) buffer, pH 3 just prior to reversed phase HPLC chromatography, as shown in Figure S2.

#### Reversed phase chromatography

In order to allow loading of the sample in aqueous solution we had to use a C-18 reversed phase matrix with polar end-capping (Aquasil). As shown below (Figure S2) the activity was very weakly retained by the column.

The recovery of the activity was reasonable (55–60 %) after the Aquasil HPLC step (Table S2).

Table S2 Recovery of the M44 cell death inducing activity in the different steps of the bio-guided isolation procedure. The results are given as mean ± SEM, n = 3.

**Table:** 

Bioactive fraction	Recovery (%)
Water extract supernatant	100
Water-methanol phase	96 ± 4.2
SPE flow through fraction	88 ± 2.5
RP-HPLC fraction (2.2–3.8min)	58 ± 2.2

The HPLC peak containing the apoptosis-inducing substance (Figure S2) was not homogeneous, as it eluted only slightly after the flow through fraction. The inhomogeneity was evidenced by further HPLC chromatography on various column systems, including hydrophilic interaction chromatography (HILIC), which resulted in several resolved peaks. Unfortunately, most of the bioactivity was lost during these procedures, which blocked efforts for chemical characterisation of the anti-AML activity in the M44 extract.
